# HoPaCI-DB: host-*Pseudomonas* and *Coxiella* interaction database

**DOI:** 10.1093/nar/gkt925

**Published:** 2013-10-16

**Authors:** Sophie Bleves, Irmtraud Dunger, Mathias C. Walter, Dimitrios Frangoulidis, Gabi Kastenmüller, Romé Voulhoux, Andreas Ruepp

**Affiliations:** ^1^CNRS/Aix-Marseille University, Laboratoire d’Ingénierie des Systèmes Macromoléculaires (UMR7255), Institut de Microbiologie de la Méditerranée (IMM), 31 Chemin Joseph Aiguier, 13402 Marseille cedex 20, France, ^2^Institute for Bioinformatics and Systems Biology (MIPS), Helmholtz Zentrum München - German Research Center for Environmental Health (GmbH), Ingolstädter Landstr. 1, D-85764 Neuherberg, Germany, ^3^Department of Genome-Oriented Bioinformatics, Center of Life and Food Science Weihenstephan, Technische Universität München, Freising, Germany and ^4^Bundeswehr Institute of Microbiology, Neuherbergstrasse 11, 80937 Munich, Germany

## Abstract

Bacterial infectious diseases are the result of multifactorial processes affected by the interplay between virulence factors and host targets. The host-*Pseudomonas* and *Coxiella* interaction database (HoPaCI-DB) is a publicly available manually curated integrative database (http://mips.helmholtz-muenchen.de/HoPaCI/) of host–pathogen interaction data from *Pseudomonas aeruginosa* and *Coxiella burnetii*. The resource provides structured information on 3585 experimentally validated interactions between molecules, bioprocesses and cellular structures extracted from the scientific literature. Systematic annotation and interactive graphical representation of disease networks make HoPaCI-DB a versatile knowledge base for biologists and network biology approaches.

## INTRODUCTION

After the discovery of penicillin in 1928 by Alexander Fleming and its use in medicine in the 1940s, antibiotics were considered as the most powerful weapon in the war against bacteria and referred as ‘magic bullets’. Antibiotics rapidly revolutionized the treatment of infectious diseases, but within a few years, penicillin-resistant bacteria began to appear. The enemy was fighting back, till the appearance of ‘superbugs’ that have become resistant to not just one, but to many of the commonly used antibiotics. It became evident that to disarm bacteria we must first learn more about them. Perhaps the most exciting recent advance has been the development of the interface discipline named ‘cellular microbiology’ (1), which reveals how pathogenic bacteria interact with host cells in what is turning out to be a complex evolutionary battle of competing gene products. Pathogenesis is a multifactorial process, which depends on the immune status of the host, the nature of the species or strains (virulence factors) and the number of organisms in the initial exposure. This communication occurs at many levels: colonization (mainly adherence), penetration and spread, survival in the host and tissue injury. A successful infection depends on the interplay between virulence factors and host targets as cellular components (e.g. cytoskeleton), and signalling pathways leading to inflammation or apoptosis. It is also a multidimensional process, as the programme of events is organized in time and space and the virulence factors can be switched on and off by complex regulatory networks. This cross-talk between the pathogen and its host is tightly regulated on both sides. Both players in the disease game have developed a variety of mechanisms to counter the others’ defences.

**Figure 1.**
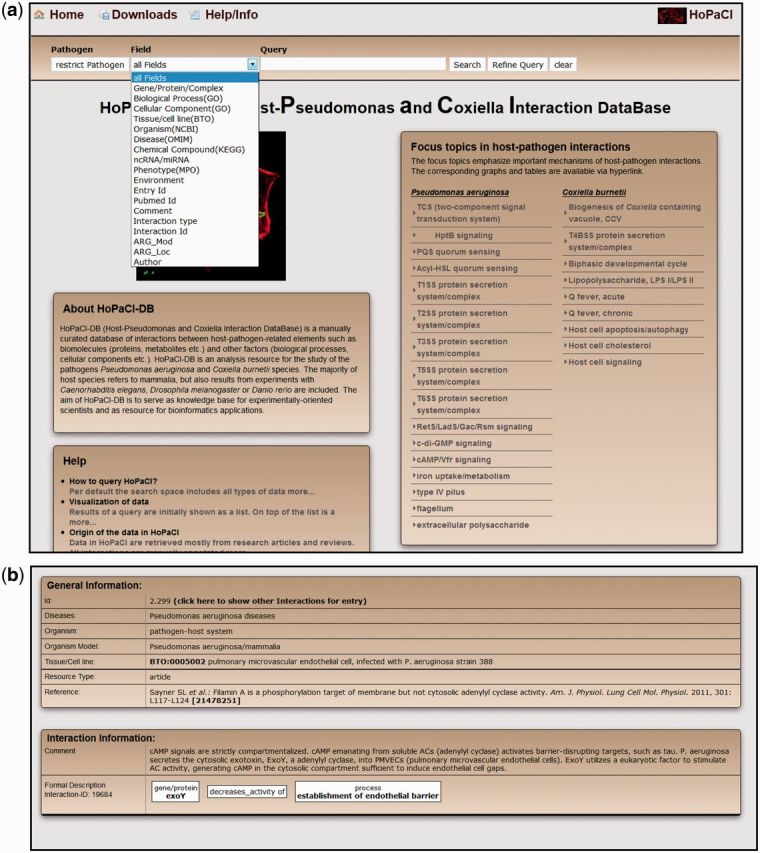
The HoPaCI-DB home page and curation of an interaction. (**a**) The HoPaCI-DB home page contains statistics, search options and links to focus topics. (**b**) A manually curated interaction containing general information, textual information (comment) and structured information (formal description).

**Figure 2.**
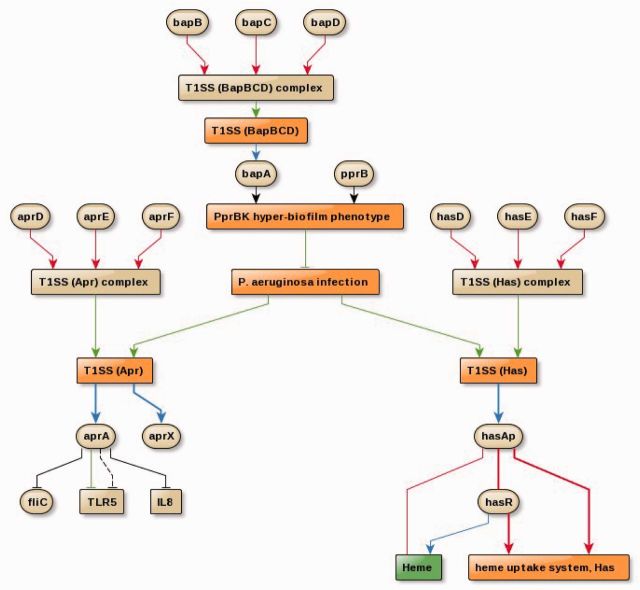
Graphical presentation of *P. aeruginosa* T1SS protein secretion system/complex focus topic. The graph shows the T1SS protein secretion system/complex with functional interactions between proteins/protein complexes (beige), chemical compounds (green) and biological processes (orange). The T1SS secretion system consists of the Apr, Has and Bap systems. T1SS (Apr) was found to be specific for the alkaline protease AprA and an uncharacterized protein (AprX). The T1SS (Has) is associated with the heme uptake system (Has), playing a role in iron utilization. T1SS (Bap) manages the transport of BapA, an adhesin involved in the two-component system PprAB triggered hyper-biofilm phenotype.

Existing information resources in the field of host–pathogen interactions cover a broad spectrum of organisms including virus-specific databases (2), bacteria-centric databases (3), fungi (4) and databases with a mixed spectrum of organisms (5,6). However, there is a lack of resources that collect experimentally verified results to annotate comprehensive biological networks on the level of cellular systems that are at the centre of the host–pathogen interactions. Here, we present HoPaCI-DB, a database with manually curated information from host–pathogen interactions. HoPaCI-DB has been developed to facilitate systems-level analyses for providing better insight into the complex networks of pathways and interactions that govern epidemiology in *Pseudomonas aeruginosa* and *Coxiella burnetii*. Multiple search options and interactive graphical presentation of networks (Figures 1 and 2) enable inspection of the manifold interrelations between heterogeneous disease factors that are required for understanding the pathobiology of acute and chronic infections.

### Curation of host–pathogen interactions

HoPaCI-DB covers host–pathogen interaction data from the pathogenic bacteria *P. aeruginosa* and *C. burnetii*. *P. aeruginosa* is an efficient Gram-negative opportunistic pathogen causing serious acute infections in patients who are immunocompromised (chemotherapy, HIV infection) or who are mechanically ventilated for instance. One in ten hospital-acquired infections is from *Pseudomonas*. *P. aeruginosa* is also responsible for fatal chronic respiratory disease of patients with cystic fibrosis. *C. burnetti* is an obligate intracellular Gram-negative zoonotic pathogen, which persists in a large reservoir among multiple species (e.g. small ruminants like sheep, cattle, goats), regularly leading to disease outbreaks (7). In humans the agent causes Q (Query) fever, which mainly is an acute disease (pneumonia, hepatitis), but in up to 2% a chronic course of the disease is seen (endocarditis) that could be fatal. The infection results from inhalation or from direct contact with milk, urine, faeces or birth products of infected animals. Because of its highly infectious nature, its high stability in the environment and its inhalational route of transmission, *C. burnetii* is recognized as a potential agent of bioterrorism.

Understanding of the pathogenic mechanisms comprises not only interrelations between pathogenic bacteria and host organisms but also intra-bacterial processes such as signalling transduction and host internal processes like inflammation. The vast majority of experimental results that have been gained in the field in recent decades are hidden in the prose of scientific literature. A comprehensive understanding of the disease-related processes requires the compilation of the distributed information in a single resource. Furthermore, generation of a resource that allows further processing of the data to perform systems biology analyses, bioinformatics analyses or graphical representation requires systematic presentation of the information and biocuration by using established biological vocabularies. Systematic transformation of complex information such as functional annotation from free text into biological vocabularies is a non-trivial task, and it has been demonstrated that current text mining methods are not yet able to produce satisfactory results for the extraction of biological information (8). The term ‘induction of endothelial cell gaps’ (9), for example, cannot easily be transferred into the respective Gene Ontology term ‘establishment of endothelial barrier’ (GO:0061028) by automated methods.

Comprehensive information extraction from the biomedical publications with high quality of the complete database content of HoPaCI-DB is obtained by experienced biocurators who manually annotate the complete articles from peer-reviewed scientific literature. The detailed manual curation permitted us to richly annotate the interactions and to place them in their relevant context. This contextual annotation includes details like the bacterial strains used in the experiments, use of host-model organisms, supporting publication, cell type, cell line and tissue. In contrast to genome-centric resources, HoPaCI-DB pursues a network-oriented approach. Host–pathogen interactions depend on a number of complex processes such as two-component signal transduction systems, quorum sensing and iron acquisition. To provide users an instructive overview about the most important mechanisms, we compiled 25 focus topics on the homepage so far. Focus topics include networks of the responsible disease-relevant factors such as proteins, protein complexes, cellular processes, chemical compounds or cellular compartments. Focus topics (see later in text) are hyperlinked to respective web pages where users can inspect lists of the involved interactions, statistics about the involved components and links to interactive graphical diagrams (Figure 2).

The biological contents offer a meaningful synopsis of the pathobiological interaction network. The focus topic ‘Acyl-HSL quorum-sensing’ (QS), for example, illustrates several disease-associated processes in *P. aeruginosa*: (i) types II, III and VI secretion systems (T2/3/6SS) are inversely controlled by QS, T2SS and T6SS (loci 2 and 3) are activated while T3SS and T6SS (locus 1) are repressed. (ii) Additional interactions show the enzymatic activities that regulate the amount of Acyl-HSL QS signalling molecules. (iii) QS regulates biofilm formation and architecture and many other virulence factors like siderophores involved in iron acquisition.

The *C. burnetii* focus topic type IVB secretion system (T4BSS) demonstrates the current model of the Icm/Dot T4BSS, shown to translocate a large number of bacterial effector proteins into the host cell during infection (Supplementary Figure S1). The graphical network figure reveals the following: (i) the constituting genes of the secretion machinery itself, (ii) the translocated effector proteins and their localization, (iii) the contribution of the phagosome acidification on the effector secretion and (iv) the impact of the T4BSS on phagosome maturation, *C. burnetii* replication and host cell death.

It should be noted that focus topics are not encapsulated entities of information but can be extended with tools offered by the graph viewer (see later in text). It is a conceptual decision to preferentially annotate literature information that can be extended to larger network structures. As of July 2013, we have reviewed 218 publications and curated 3585 disease-relevant interactions.

### Data structure of HoPaCI-DB

For transformation of the biomedical information into a data structure fulfilling the needs of wet lab scientists as well as for bioinformatics applications, information in HoPaCI-DB is structured as three types of information (10): (i) structured information, (ii) textual comment and (iii) general information.

(i) Core element for biocuration of host–pathogen interactions is the structured information describing the interaction between two elements, for example, between the compound Psl polysaccharide and the phagocytosis-associated bioprocess opsonization (see interaction-ID: 30776). Subjects and objects are molecules such as proteins, nucleic acids or chemical compounds and other elements like cellular processes, phenotypes or environmental factors. To provide the content of HoPaCI-DB in a standardized format, we use names and identifiers from established resources like EntrezGene (11), KEGG (12) or CORUM (13) for annotation. (ii) The very basic information of the structural part is complemented by the textual comment. This part provides information concerning experimental conditions, details about the infection process or exact cellular localization of a process. As an example, the *modus operandi* how *P. aeruginosa* injects a soluble adenylate cyclase, ExoY, into the cytosol of pulmonary microvascular endothelial cells generating a cAMP signal that disrupts the endothelial cell barrier is not representable by a structured information but requires a textual comment (Interaction-ID: 19684). (iii) The general information includes basic contents such as literature reference or host and pathogen organisms and also details like bacterial strain, organism model, tissue and cell line. Most studies use the *Pseudomonas* strains PAO1 or PA14. The increased virulence of PA14 is mainly due to ExoU, a type III secreted potent cytotoxin, absent in PAO1, and a mutation in the *ladS* gene that leads to an elevated T3SS activity and increased cytotoxicity towards mammalian cells (14).

Host–pathogen interaction information is enriched with data from external resources. Whenever possible, elements from HoPaCI-DB are hyperlinked to information-rich database entries from PubMed (15), EntrezGene (11), KEGG (16), Gene Ontology (17) and other resources. The complete database information or results from database searches can be downloaded as flat files or in Systems Biology Markup Language (SBML), a free and open interchange XML format (18). Files in the SBML format can be visualized and analysed with network analysis tools such as Cytoscape (19).

Other publicly available resources that provide host–pathogen interaction information are rather gene or genome oriented. PATRIC is a bacterial bioinformatics resource with a focus on human pathogenic species (3). PATRIC includes a built-in system for predicting genes, assigning gene functions and reconstructing metabolic pathways. The resource links the database information to a variety of external resources such as KEGG, Gene Ontology and PDB and offers various analysis tools. PHI-base has an even broader scope by providing manually curated information of pathogenicity, virulence and effector genes from fungal, oomycete and bacterial pathogens, which infect animal, plant, fungal and insect hosts (5). PHI-base catalogues validated genes that are required for the disease-causing ability of a microbe, genes of the host response and verified targets of known bioactive compounds, which either kill pathogens or arrest pathogen growth/development. Resources such as PATRIC or PHI-base provide a wealth of details that complement the network-oriented approach of HoPaCI-DB.

### Search options and visualization

In addition to the predefined focus topics, flexible web-based interface of HoPaCI-DB allows to investigate an area of interest by various search options (Figure 1). The basic search covers all annotated information from HoPaCI-DB or can be restricted for specific types of information such as ‘gene/protein/complex’, ‘biological process’ or ‘chemical compound’. To make the search operation intuitive, all of the information types are listed in a ‘dropdown box’. If results of an initial search need refinement, HoPaCI-DB offers addition of further searches by using the ‘refine query’ option. Extension or contraction of the search space can be achieved by using one of the three operators ‘and’, ‘or’ and ‘not’. The search results appear as a list that is linked to entries providing the details of the manually curated information.

In addition, search results are linked to a graphical tool that dynamically generates a graph from the search results (Figure 2). Within the interactive graph, interaction information is shown as colour-coded nodes (nodes are objects such as a protein, chemical compound or bioprocesses). The nodes are linked via edges defining the mode of interaction (e.g. protein A increases_activity of protein B). The graph software offers tools for convenient retrieval of the annotated information: (i) While moving the mouse cursor over edges, pop-up windows appear that present the user important information of the interaction such as the comment and the literature reference. (ii) The graph tool allows choosing between two different options, organic or hierarchical layout. (iii) Other functionalities include options to move nodes within the graph or to extend the graph with all interaction information about a node of interest. A description of the different functionalities can be found on the help pages of HoPaCI-DB.

### Application of HoPaCI-DB

As said before, *P. aeruginosa* causes acute and chronic infections. The choice between those two modes of virulence and life styles is under the control of a complex regulatory network, involving notably two small RNAs, RsmY and RsmZ. Through their effect on the post-transcriptional RsmA regulator, they are controlling key virulence factors as flagellum, type IV pili, biofilm, T3- and T6SS. The expression of these two small RNAs is crucial for the attenuated persistence of *P. aerugniosa* in lungs of infected people. The fine interconnection between this cascade of regulators and *P. aeruginosa* virulence factors is now easily accessible to a broader audience, thanks to the graphical view of the HoPaCI-DB (Supplementary Figure S2). This representation, which highlights the central role of these small RNAs, constitutes a schematic picture and reveals new targets for the development of antibacterials. Looking for inhibitors of RsmY and RsmZ expression can be an alternative strategy to the use of antibiotics to fight the infection.

Moreover, the HoPaCI-DB graphical view presentation provides a comprehensive overview on the architectural organization of the different *Pseudomonas* nanomachines involved in protein secretion. The T2SS, for example, is constituted by at least 12 different Xcp proteins. The Xcp proteins are recovered in both bacterial membranes and interact with each other in three sub-complexes: an inner membrane platform, an outer membrane pore and a transperiplasmic pilus-like structure. By clicking on the Xcp T2SS graphical view representation in HoPaCI-DB, we have immediate access to all the interactions so far discovered among the different components and their sub-organization in the bacterial envelope. This unprecedented representation opens the search for new protein–protein interactions among those components. More interestingly, this can be extended to the secreted substrates to explore the sequential interactions of the substrates with the machinery during the transport. Deciphering the assembly mechanism and the T2SS/substrate interactions may make it possible to identify new targets for the development of antimicrobial disruptors.

To date, little is known about *C. burnetii* virulence factors. One established observation is that *Coxiella* is present in a specific cellular niche of the host: the phagolysosome. Several studies in the recent years revealed different parts of the regulatory network, which facilitates the survival of *Coxiella* under the harsh conditions of the phagolysosome. Using the HoPaCI-DB, it is possible to present these parts and its (transitive) connections in an interactive manner. As an example, we executed the simple query ‘phagosome maturation’ to give an overview of the different cellular components and biological processes involved in this process (Supplementary Figure S3). The complex structure is presented comprehensibly in a graphical manner (based on 39 interactions extracted from 15 articles). Phagosome maturation triggered by *C. burnetii* infection is increased by several pathway structures like acidification, endocytosis, actin cytoskeleton reorganization and Dot/Icm type IVB T4BSS. It finally ends up in the generation of a special phagolysosome, the parasitophorous vacuole. Inside this structure, both variants of *C. burnetii* (phase I and phase II) are able to replicate, demonstrating that the only known virulence factor LPS is, therefore, not responsible for the survival inside the parasitophorous vacuole.

Using the HoPaCI-DB, it is also possible to show interactions of drugs and chemical compounds, which offer the opportunity to identify new aspects in the treatment and prophylaxis of Q fever. For example, in Supplementary Figure S3, chloramphenicol is involved in the generation of the phagolysosome because it is able to block the fusion of endocytic vesicles and lysosomes and the formation of the characteristic large spacious vacuole. Another compound, the antiviral substance and protein inhibitor Brefeldin A, is able to decrease the size of the *Coxiella* replicative vacuoles. Even the information presented here is based on a limited number of publications; it shows the advantages and strength of such an integrative view of numerous and heterogeneous data. In the future, we will update the database annually and provide novel host–pathogen interactions for HoPaCI-DB.

## SUPPLEMENTARY DATA

Supplementary Data are available at NAR Online.

## FUNDING

Funding for open access charge: The “Pathomics” ERA-net PATHO Grant [ANR-08-PATH-004-01] supported this work (in part). Helmholtz Zentrum München - German Research Center for Environmental Health (GmbH); The German Ministry of Education and Research (BMBF) under contract No. [01KI1001] supported this work (in part).

*Conflict of interest statement*. None declared.
